# Developmental Expression and Glucocorticoid Control of the Leptin Receptor in Fetal Ovine Lung

**DOI:** 10.1371/journal.pone.0136115

**Published:** 2015-08-19

**Authors:** Miles J. De Blasio, Maria Boije, Owen R. Vaughan, Brett S. Bernstein, Katie L. Davies, Alice Plein, Sarah L. Kempster, Gordon C. S. Smith, D. Stephen Charnock-Jones, Dominique Blache, F. B. Peter Wooding, Dino A. Giussani, Abigail L. Fowden, Alison J. Forhead

**Affiliations:** 1 Department of Physiology, Development and Neuroscience, University of Cambridge, Downing Street, Cambridge, United Kingdom; 2 Department of Medicine, University of Cambridge, Addenbrooke’s Hospital, Cambridge, United Kingdom; 3 Department of Obstetrics and Gynaecology, University of Cambridge, The Rosie Hospital, Cambridge, United Kingdom; 4 School of Animal Biology, University of Western Australia, Perth, Western Australia, Australia; 5 Department of Biological and Medical Sciences, Oxford Brookes University, Oxford, United Kingdom; University of Giessen Lung Center, GERMANY

## Abstract

The effects of endogenous and synthetic glucocorticoids on fetal lung maturation are well-established, although the role of leptin in lung development before birth is unclear. This study examined mRNA and protein levels of the signalling long-form leptin receptor (Ob-Rb) in fetal ovine lungs towards term, and after experimental manipulation of glucocorticoid levels *in utero* by fetal cortisol infusion or maternal dexamethasone treatment. In fetal ovine lungs, Ob-Rb protein was localised to bronchiolar epithelium, bronchial cartilage, vascular endothelium, alveolar macrophages and type II pneumocytes. Pulmonary Ob-Rb mRNA abundance increased between 100 (0.69 fractional gestational age) and 144 days (0.99) of gestation, and by 2–4-fold in response to fetal cortisol infusion and maternal dexamethasone treatment. In contrast, pulmonary Ob-Rb protein levels decreased near term and were halved by glucocorticoid treatment, without any significant change in phosphorylated signal transducer and activator of transcription-3 (pSTAT3) at Ser727, total STAT3 or the pulmonary pSTAT3:STAT3 ratio. Leptin mRNA was undetectable in fetal ovine lungs at the gestational ages studied. These findings demonstrate differential control of pulmonary Ob-Rb transcript abundance and protein translation, and/or post-translational processing, by glucocorticoids *in utero*. Localisation of Ob-Rb in the fetal ovine lungs, including alveolar type II pneumocytes, suggests a role for leptin signalling in the control of lung growth and maturation before birth.

## Introduction

Since identification of the obese (ob) gene and its protein product in 1994, and the leptin receptor (Ob-R) in the following year, the physiological functions of leptin in adult life have been extensively studied and well-characterised [1, 2, 3]. In adult animals, leptin is a polypeptide hormone primarily synthesised and secreted by white adipocytes. It has a key role as a nutritional signal in the regulation of energy intake and expenditure, and in the maintenance of reproductive and immune function [1, 4]. Leptin acts on transmembrane receptors in target cells and there are at least six splice variants of the leptin receptor gene [5]. The long-form leptin receptor Ob-Rb, however, is the only receptor with the cytoplasmic signalling domain to fully activate a variety of intracellular pathways, including the Janus kinase (JAK) and signal transducers and activators of transcription (STAT) pathway [5].

In contrast to the adult, relatively little is known about the developmental regulation and role of leptin in the fetus. In many animal species, including human and sheep, leptin is detected in the fetal circulation from mid-gestation, and there is widespread expression of leptin receptors in fetal and placental tissues [6]. In the sheep fetus, there is a rise in adipose leptin mRNA and plasma leptin levels with gestational age, coincident with the pre-partum surges in plasma cortisol and triiodothyronine (T_3_), and which can be abolished by fetal adrenalectomy and stimulated prematurely in response to endogenous and synthetic glucocorticoid treatment [7, 8, 9]. Furthermore, synthetic glucocorticoids such as dexamethasone, administered to pregnant women at risk of preterm delivery, cause an increase in umbilical cord blood leptin concentrations in the neonate [10].

The rise in cortisol in the fetal circulation near term is known to have maturational effects in the fetus that are essential for the successful transition to the extrauterine environment at birth [11]. In particular, cortisol promotes pulmonary maturation *in utero* and normal respiratory function at delivery. Antenatal synthetic glucocorticoids aid fetal lung maturation, prevent respiratory distress and improve survival and health outcomes of the premature human infant [12]. Endogenous and synthetic glucocorticoids stimulate maturation of a variety of structural aspects in the fetal lungs, including alveolarisation, thinning of the alveolar septae and increased pulmonary collagen and elastin content [13]. They also activate synthesis of both the lipid and protein components of surfactant and its secretion from type II pneumocytes in the alveoli [14]. Expression of leptin and leptin receptors has been demonstrated in the fetal lungs of rodent and non-human primate species [15, 16, 17, 18]. However, the extent to which the maturational effects of glucocorticoids on the fetal lung may be mediated, at least in part, by leptin is unknown. This study examined the mRNA and protein content of leptin and its long-form receptor (Ob-Rb), and the phosphorylation status of STAT3, in fetal ovine lungs during late gestation, and after experimental manipulation of glucocorticoids by fetal cortisol infusion or by maternal dexamethasone treatment. The study hypothesised that pulmonary Ob-Rb mRNA and protein levels, and JAK/STAT signalling, would increase in the sheep fetus towards term and in response to glucocorticoid exposure *in utero*.

## Materials and Methods

### Animals

All surgical and experimental procedures were carried out in accordance with the UK Animals (Scientific Procedures) Act 1986 and were approved by the animal ethics committee of the University of Cambridge. Time-dated pregnant Welsh Mountain ewes were housed in individual pens in sight of other ewes, and fed concentrates (200 g/d; 18% protein and 10 MJ/kg; Sheep Nuts #6; H&G Beart, Kings Lynn, UK) with hay, water and a salt-lick block *ad libitum*. Overall, the animals consumed between 8–11 MJ/d of metabolisable energy.

### Surgical and experimental procedures

#### Developmental study

Twenty ovine fetuses were examined at 100, 115, 130 and 144 days (d) of gestation (n = 5 per age group, term approximately 145d). Each group contained a mixture of twin and singleton fetuses.

#### Fetal cortisol study

Under maternal halothane anaesthesia (1.5% in O_2_-N_2_O), catheters were implanted into the femoral artery and vein of eleven singleton fetuses at approximately 116d of gestation using surgical methods described previously [19]. Food, but not water, was withheld from the pregnant ewe for 18–24h before surgery. After surgery, fetal and maternal blood samples (2 ml) were collected daily to monitor maternal and fetal blood gas status. At least six days after catheterisation, the fetuses were infused intravenously with either cortisol (2–3 mg/kg/d in 3.0 ml 0.9% saline; EF-Cortelan, GlaxoSmithKline, Brentford, UK; n = 5) or saline vehicle (3.0 ml/d 0.9% wt/vol; n = 6) for five days from 125d of gestation. The cortisol dose administered achieves plasma cortisol concentrations observed in fetal sheep near term without inducing parturition [11]. Tissues were collected at 130d of gestation.

#### Maternal dexamethasone study

Eleven pregnant ewes carrying singleton fetuses were injected twice intramuscularly with either dexamethasone (12 mg dexamethasone sodium phosphate; Merck Sharpe and Dohme Ltd, Hoddesdon, UK; n = 5) in 2 ml saline or saline (0.9% w/v NaCl; n = 5) at 24h intervals from 125d of gestation. Tissues were collected 10h after the second maternal injection on 127d of gestation. The dose of dexamethasone administered is similar to that recommended for clinical use in women threatening with premature delivery [20].

#### Blood and tissue collection

The fetuses from all studies were delivered by Caesarean section with the ewe under general anaesthesia (20 mg/kg sodium pentobarbitone i.v.). At delivery, an umbilical arterial blood sample (10 ml) was collected, and the fetus and ewe were euthanized by barbiturate (200 mg/kg sodium pentobarbitone). The fetal lungs were weighed and un-inflated samples were fixed in 4% paraformaldehyde (with 0.2% gluteraldehyde in 0.1M phosphate buffer, pH 7.4) and embedded in paraffin wax for histological analysis. Samples of fetal lung were also frozen in liquid nitrogen and stored at -80°C for biochemical and molecular analyses. All blood samples collected in the studies were placed into EDTA-containing tubes and centrifuged at 1000x*g* and 4°C for 5 min. The plasma samples were stored at -20°C until analysis.

### Biochemical and molecular analyses

#### Plasma hormone analyses

All radioimmunoassay (RIA) methods were validated for use with ovine plasma. Plasma leptin concentrations were measured by in-house RIA using ovine leptin standards as previously described [7, 21]; the lower limit of detection was 0.09 ng/ml and the inter-assay coefficient of variation was 5%. Plasma cortisol concentrations were measured by in-house RIA [22] where the lower limit of detection was 1.0–1.5 ng/ml and the inter-assay coefficient of variation was 12%. Total plasma T_3_ and thyroxine (T_4_) concentrations were also measured by RIA using a commercial kit [23] (ICN Biomedicals, Thame, UK). The lower limits of detection were 0.07 ng/ml for T_3_ and 7.6 ng/ml for T_4_, and the inter-assay coefficients of variation were 10% for both assays.

#### Immunohistochemistry

Sections (5μm) of fetal lung were cut and mounted. Sections were de-waxed and rehydrated in xylene and graded percentages of industrial methylated spirit (100–50% IMS). The sections were incubated in 3% hydrogen peroxide for 15 min to remove endogenous peroxidases. A heat-activated antigen retrieval stage was carried out by incubating the slides in 10mM citric buffer (pH 6) in a microwave pressure cooker for 2 min at 800W. This was followed by a 1h incubation in a humidity chamber with 5% goat serum in 2% bovine serum albumin and phosphate-buffered saline (0.01M PBS, pH 7.4; Sigma, Poole, UK) to reduce non-specific antibody binding. The sections were incubated with a rabbit polyclonal antibody to the C-terminus of the long-form ovine leptin receptor (1:500 dilution of 0.5 mg/ml; orb6312, Biorbyt, Cambridge, UK) overnight in a humidity chamber at 4°C. Negative control samples were incubated in the diluent (5% goat serum in PBS). Protein binding was detected with a biotinylated goat anti-rabbit secondary antibody using the Vectastain Elite ABC system (Vector Laboratories, Peterborough, UK) and diaminobenzidine (Sigma, Poole, UK) according to the manufacturers’ instructions. The sections were counter-stained with methyl green (Vector Laboratories, Peterborough, UK) at 60°C for 3 min. Slides were scanned using a NanoZoomer system (Hamamatsu, Hamamatsu City, Japan), and viewed using Nanozoomer Digital Pathology (NDP) view software.

#### Quantitative RT-PCR

Frozen samples of fetal lung tissue (15mg) were placed in Lysing Matrix-D tubes (MP Biomedicals, Loughborough, UK) with 170 μl lysis/binding solution from a MagMax96 Total RNA Isolation kit (Life Technologies, Paisley, UK) and 0.75 μl β-mercaptoethanol, and homogenized using a FastPrep-24 (MP Biomedicals, Santa Ana, USA). After homogenisation, 106 μl 100% isopropanol was added to each sample. Samples were placed into a MagMAX96 system (Applied Biosystems, Paisley, UK) where RNA was isolated and DNase treated (TURBO DNase) using the MagMAX96 Total RNA Isolation Kit (Life Technologies). Sample RNA yields and purities were assessed by a Nanodrop (Thermo Fisher, Loughborough, UK). Ratios of absorption (260/280nm) of all preparations were between 1.8 and 2.0.

Reverse transcription of mRNA was carried out using a PCR Express machine (Thermo Fisher) and materials from Promega (Southampton, UK) and Invitrogen (Paisley, UK). For each sample, 5 μl of DNAse-treated RNA was mixed with 1μl random primers, 1μl deoxyribonucleotide triphosphate mix and 5 μl RNAse-free water, and incubated at 65°C for 2 min. A master reverse transcription mix was made, consisting of 4 μl first strand buffer, 2 μl dithiothreitol, 1 μl RNAseOUT and 1 μl Superscript II enzyme. The samples were incubated at room temperature for 5 min, at 42°C for 50 min and at 70°C for 15 min.

TaqMan qRT-PCR was performed to measure mRNA abundance of target genes in lung samples. Samples were analysed using a TaqMan 7900HT and data were acquired and processed with Sequence Detector v.2.3 software (Applied Biosystems). TaqMan Master Mix (5 μl), 0.5 μl target gene probe and primer set, and 3.5 μl water, were added to each well of a 96-well HT plate (Applied Biosystems). In addition, 1 μl lung cDNA at 1:20 dilution was added to each well apart from the non-template controls, where 1 μl of water was added. The sequences of the TaqMan qRT-PCR probes for leptin, all forms of the leptin receptor (Ob-R) and the functional long form of the leptin receptor (Ob-Rb) are listed in Table 1. Each sample was measured in triplicate and normalised to the geometric mean of two housekeeping genes, GAPDH and cyclophilin A (Table 1) [24, 25]. For each plate, a negative control without cDNA was used to ensure that no amplicon contamination had occurred in the reaction. In addition, one cDNA sample was measured on each plate across all assays as a quality control. In order to compare mRNA abundance of target genes, cycle thresholds (Ct) determined by qRT-PCR were analysed via the ddCt method as all standard curves were linear and parallel.

**Table 1:** Primer and reporter sequences used for TaqMan qRT-PCR in the sheep.

Gene	Forward Primer Sequence	Reverse Primer Sequence	Reporter Sequence	Reporter dye
Leptin	CAAGACGATTGTCACCAGGATCAA	CCAGTGACCCTCTGTTTGGA	TCACACACGCAGTCCGT	FAM
Ob-Rb (long-form)	GGAGACAGCCCTCTGTTAAATATGC	TGAGCTGTTTATAAGCCCTTGCT	CCTCCTCGGCTTCACC	FAM
Ob-R (all isoforms)	GAGCGCCCTTCTTACCTTTACTA	CCAACCGCTGTCAGAATTTTAGGT	CACAAGATGTCATATATTTTC	FAM
GAPDH	GCTACACTGAGGACCAGGTT	AGCATCGAAGGTAGAAGAGTGAGT	CTCCTGCGACTTCAAC	FAM
Cyclophilin A	GGTTCCTGCTTTCACAGAATAATTCC	GTACCATTATGGCGTGTGAAGTCA	CACCCTGGCACATAAA	FAM

#### Western blotting

Frozen samples of fetal lung (100 mg) were homogenised in 1 ml ice-cold lysis buffer containing 20 mM Tris, pH 7.4, 1 mM EGTA, 0.01% Triton X-100, 1 mM sodium pyrophosphate, 1 mM sodium orthovanadate, 10 mM β-glycerol phosphate, 50 mM sodium fluoride and protease inhibitor cocktail (Roche Diagnostics, Burgess Hill, UK). Samples were centrifuged at 15,000x*g* for 10 min at 4°C. The protein concentration of the lysates was measured by a bicinchoninic acid protein assay (Sigma, Poole, UK). The samples were mixed with SDS-PAGE gel loading buffer (50 mM Tris-HCl, pH 6.8, 100 mM dithiothreitol, 2% SDS, 10% glycerol, bromophenol blue) and boiled for 5 min.

Equal amounts of sample protein (50 μg) were separated using 7.5% Mini-PROTEAN precast gels for 40 min at 200V and according to the manufacturer’s instructions (Bio-Rad Laboratories Inc., Hemel Hempstead, UK) and transferred for 1.5h at 10V onto nitrocellulose membrane (Invitrogen, Paisley, UK). The membrane was incubated with 5% non-fat dried milk in Tris-buffered saline and 0.1% Tween-20 for 1h at room temperature, followed by incubation overnight at 4°C with rabbit polyclonal primary antibodies to (a) the ovine long-form leptin receptor (Ob-Rb; 2 μg/ml orb6312, Biorbyt, Cambridge, UK), or (b) the mouse phosphorylated STAT3 containing Ser727 (0.5 μg/ml sc-8001-R, Insight Biotechnology Ltd, Wembley, UK) or (c) the human STAT3 (1:500, 07–2173, Millipore, Watford, UK). Each membrane was incubated with a horseradish peroxidase-conjugated anti-rabbit secondary antibody (1:5,000; Amersham Biosciences, Amersham, UK) for 1h at room temperature. Protein expression was visualised using the ECL Plus chemiluminescence system according to the manufacturer’s instructions (Amersham Biosciences, Amersham, UK) and quantified using ImageJ software (National Institutes of Health, MD, USA; http://rsb.info.nih.gov/ij/) after normalisation to Ponceau S staining [26]. Ratios of protein expression were arcsine-transformed before statistical analysis. The immunoblots showed bands at the expected sizes of around 103kDa (Ob-Rb) and 90kDa (pSTAT3 and STAT3). All samples from the fetal cortisol study, and the maternal dexamethasone study, were analysed on a single gel while the samples from the developmental study were analysed on two gels with a single sample assessed on each. The data from the developmental study were normalised to the quality control sample in the two gels.

### Statistical analysis

All data (**S1 File**) were analysed for normality, and parametric tests were performed on normally-distributed data while non-parametric tests were carried out on data that were not normally distributed (SPSS, Richmond, USA). Developmental data were assessed by one-way ANOVA followed by the Tukey post-hoc test, whilst data obtained from the fetal cortisol and maternal dexamethasone studies were compared by the unpaired Student’s t-test with their respective saline control groups. Pearson correlation analysis was used to determine relationships between plasma hormone (log_10_ concentration) and leptin receptor levels. Fold changes from the group mean at 100 d (developmental study) or from the control group mean (fetal cortisol and maternal dexamethasone studies) are presented as mean ± SEM. Differences where P*<*0.05 were regarded as significant.

## Results

### Fetal body and lung weights

Fetal body weight (100d: 0.76 ± 0.03 kg; 115d: 1.42 ± 0.08 kg; 130d: 2.36 ± 0.23 kg; 144d: 3.50 ± 0.22 kg; P<0.05) and lung wet weight (100d: 33.7 ± 2.9 g; 115d: 60.3 ± 1.4 g; 130d: 73.4 ± 10.0 g; 144d: 84.1 ± 13.0 g; P<0.05) increased, and the relative lung wet weight (expressed as a percentage of the body weight; 100d: 4.45 ±0.36%; 115d: 4.30 ± 0.18%; 130d: 3.07 ± 0.17%; 144d: 2.42 ±0.32%; P<0.05) decreased with advancing gestational age. Fetal cortisol infusion and maternal dexamethasone treatment did not affect fetal body weight, or absolute or relative lung weights (data not shown).

### Plasma hormone concentrations

Fetal plasma concentrations of cortisol and T_3_ increased in the untreated fetuses of the developmental study with advancing gestation (P<0.001); plasma cortisol and T_3_ were significantly greater at both 130d and 144d compared to 100d of gestation (P<0.05; Table 2). Plasma leptin concentration showed an increasing trend between 100d and 144d of gestation, but this failed to reach significance (P = 0.085; Table 2). Plasma T_4_ concentration remained unaltered over the gestational ages studied (Table 2).

**Table 2:** Mean (± SEM) plasma hormone concentrations in untreated ovine fetuses between 100 and 144d of gestation, and in glucocorticoid-treated ovine fetuses at 127–130d of gestation (n = 5–6 in each group). For the developmental study, values in each column with different superscripts are significantly different from each other (P<0.05; one-way ANOVA followed by Tukey test).

	Cortisol (ng/ml)	T_3_ (ng/ml)	T_4_ (ng/ml)	Leptin (ng/ml)
**Developmental study**				
100d (n = 5)	3.0 ± 0.6 ^a^	0.12 ± 0.02 ^a^	73.8 ± 5.0	0.78 ± 0.06
115d (n = 5)	4.4 ± 0.3 ^a^	0.15 ± 0.02 ^a^	99.6 ± 2.9	0.89 ± 0.06
130d (n = 5)	9.9 ± 1.4 ^b^	0.21 ± 0.02 ^b^	96.8 ± 10.0	0.85 ± 0.08
144d (n = 5)	59.6 ± 17.3 ^c^	0.36 ± 0.04 ^c^	72.4 ± 8.5	1.07 ± 0.11
**Fetal cortisol study**				
Saline 127–130d (n = 6)	13.4 ± 2.3	0.22 ± 0.02	98.7 ± 16.1	0.69 ± 0.03
Cortisol 127–130d (n = 5)	91.2 ± 20.9*	0.67 ± 0.07*	113.6 ± 12.2	1.06 ± 0.11*
**Maternal dexamethasone study**				
Saline 127–130d (n = 5)	16.1 ± 2.8	0.28 ± 0.06	132.5 ± 11.7	0.72 ± 0.05
Dexamethasone 127–130d (n = 5)	8.6 ± 0.7*	0.77 ± 0.05*	126.3 ± 22.0	1.04 ± 0.06*

* significantly different from respective saline control fetuses, P<0.05, unpaired t-test.

Five days of fetal cortisol infusion significantly increased plasma concentrations of cortisol, T_3_ and leptin (P<0.05) compared with fetal saline infusion, but had no effect on plasma T_4_ concentration (Table 2). Maternal dexamethasone administration increased plasma T_3_ and leptin (P<0.05), and decreased plasma cortisol (P<0.05), in the fetus compared with maternal saline treatment, but did not affect plasma T_4_ concentration (Table 2).

### Pulmonary localisation of Ob-Rb protein

**Fig 1:**
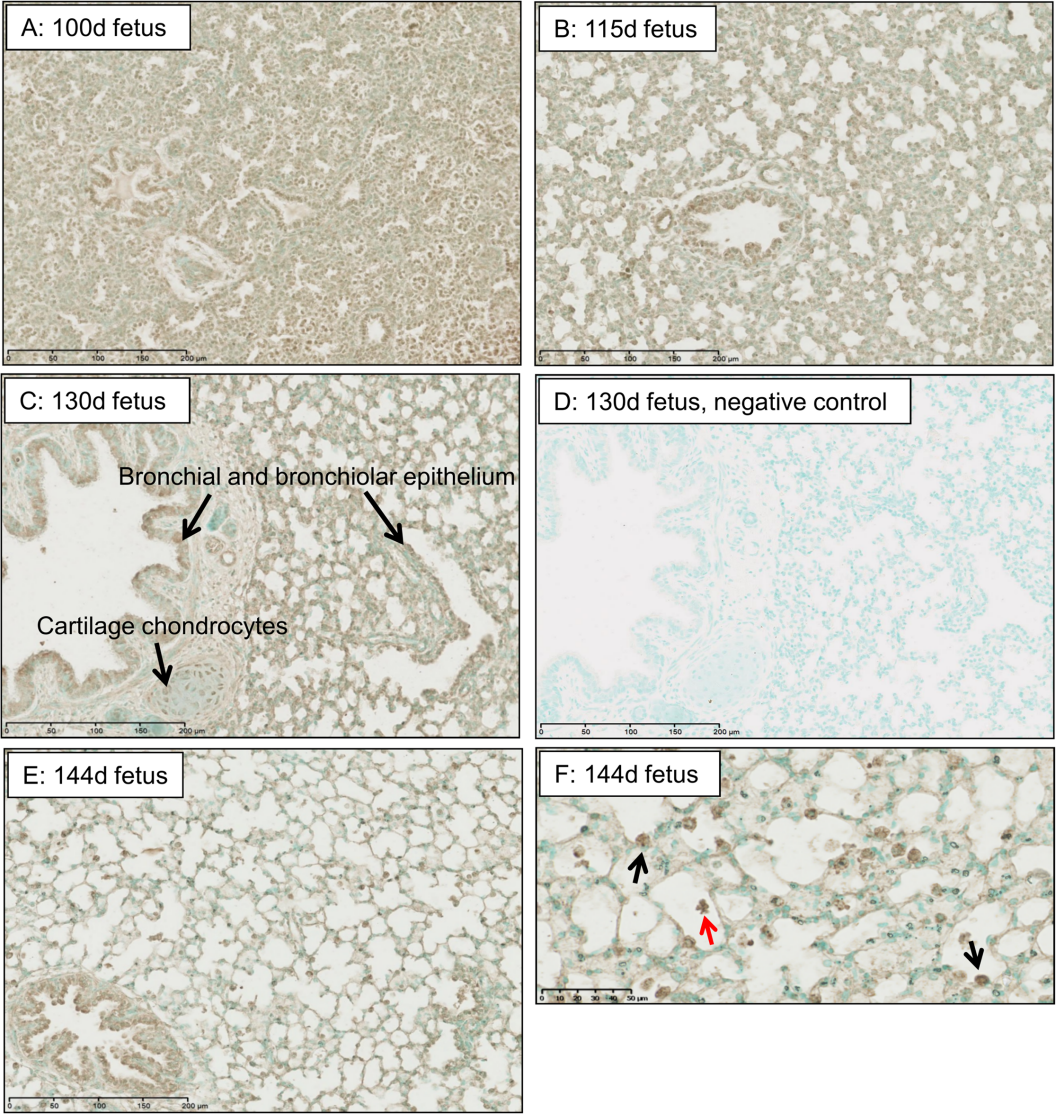
Immunohistochemical localisation of Ob-Rb in fetal ovine lungs during late gestation. Fetal lungs at 100d (A), 115d (B), 130d (C) and 144d (E, F); negative control at 130d (D). Arrowheads show leptin staining in fetal bronchial and bronchiolar epithelium, and cartilage chondrocytes (C), type II pneumocytes (F, black) and alveolar macrophages (F, red).

In the fetal ovine lungs, Ob-Rb protein was present from 100d of gestation throughout the developing pulmonary tissue, including bronchiolar epithelium (Fig 1A). By 130d of gestation, Ob-Rb protein was localised to the cells of the bronchial and bronchiolar epithelium, the chondrocytes in bronchial cartilage and the vascular endothelium (Fig 1C), and by 144d of gestation, leptin receptor staining was clearly localised to the type II pneumocytes and macrophages of the alveoli (Fig 1E and 1F).

### Pulmonary mRNA and protein levels of Ob-Rb, and JAK/STAT signalling

**Fig 2:**
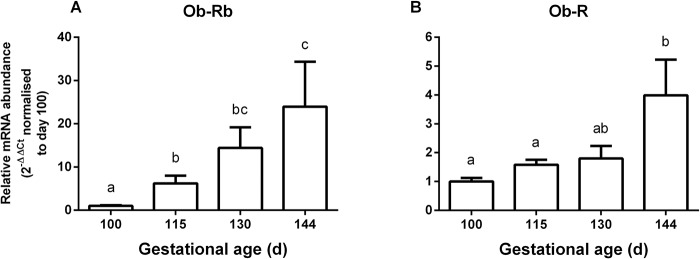
Mean (± SEM) relative mRNA abundance, normalised to 100d of gestation, of leptin receptors Ob-Rb (A) and Ob-R (B) in the lungs of sheep fetuses from 100 to 144d of gestation (n = 5 at each age). Values with different superscripts are significantly different from each other (P<0.05; one-way ANOVA followed by Tukey test).

**Fig 3:**
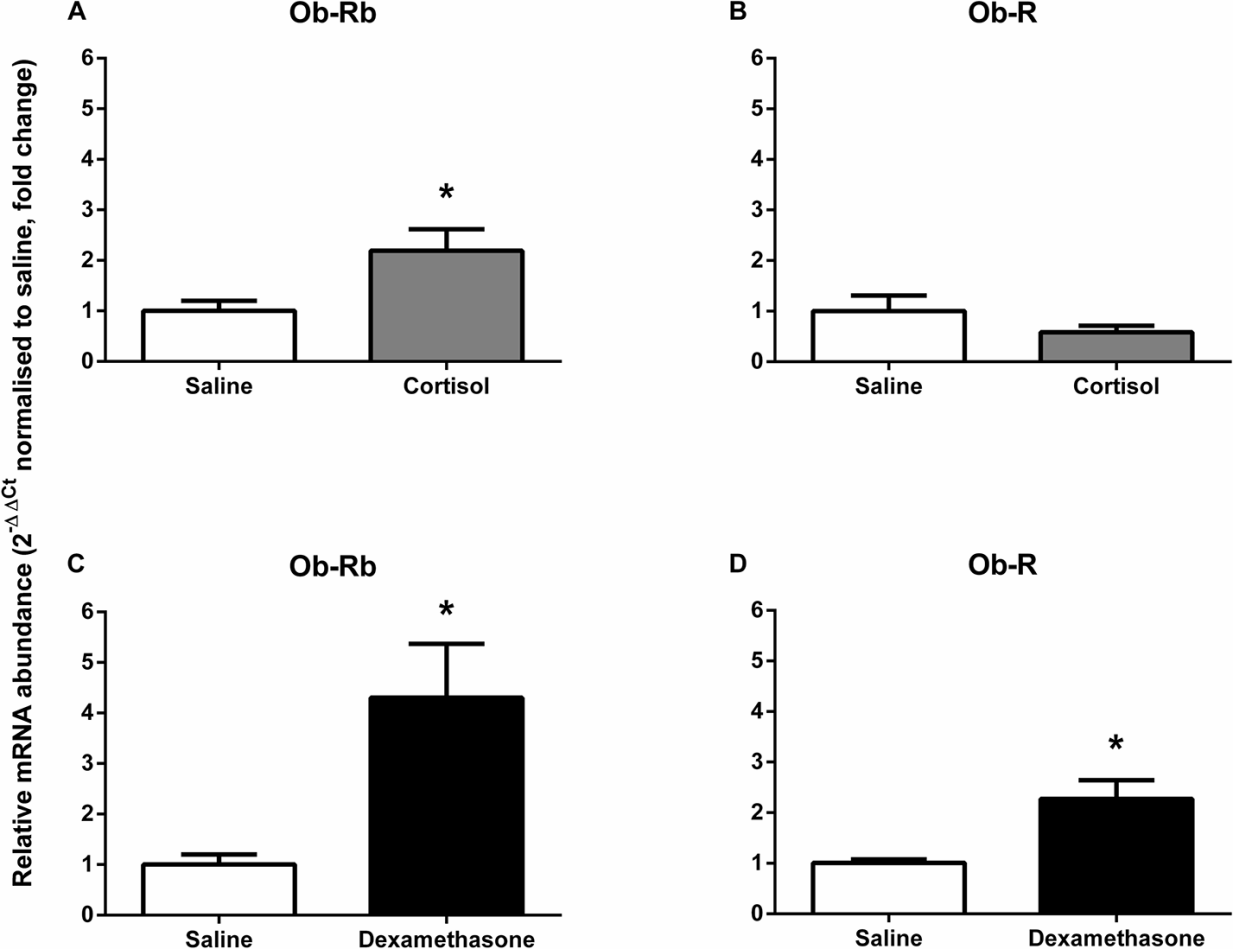
Mean (± SEM) pulmonary Ob-Rb protein levels in sheep fetuses (A) from 100 to 144d of gestation and after glucocorticoids exposure by (B) fetal cortisol infusion and (C) maternal dexamethasone treatment. For the developmental study, values with different superscripts are significantly different from each other (P<0.05; one-way ANOVA followed by Tukey test; n = 5 for each age). * significantly different from respective control saline group, P<0.05, unpaired t-test (n = 5–6 for each group).

Pulmonary leptin Ob-Rb mRNA levels showed a significant increase with gestational age (P<0.05) and was increased at 115d, 130d and 144d of gestation compared to 100d (P<0.001), and at 144d compared with 115d of gestation (P<0.05; Fig 2A). Pulmonary Ob-R (all forms) mRNA abundance also increased with advancing gestation (P<0.05) and was increased at 144d of gestation compared to 100d and 115d of gestation (P<0.05; Fig 2B). In contrast, Ob-Rb protein content significantly decreased towards term (P<0.05) and was reduced at 144d compared to 100d and 115d of gestation (P<0.05, Fig 3A).

**Fig 4:**
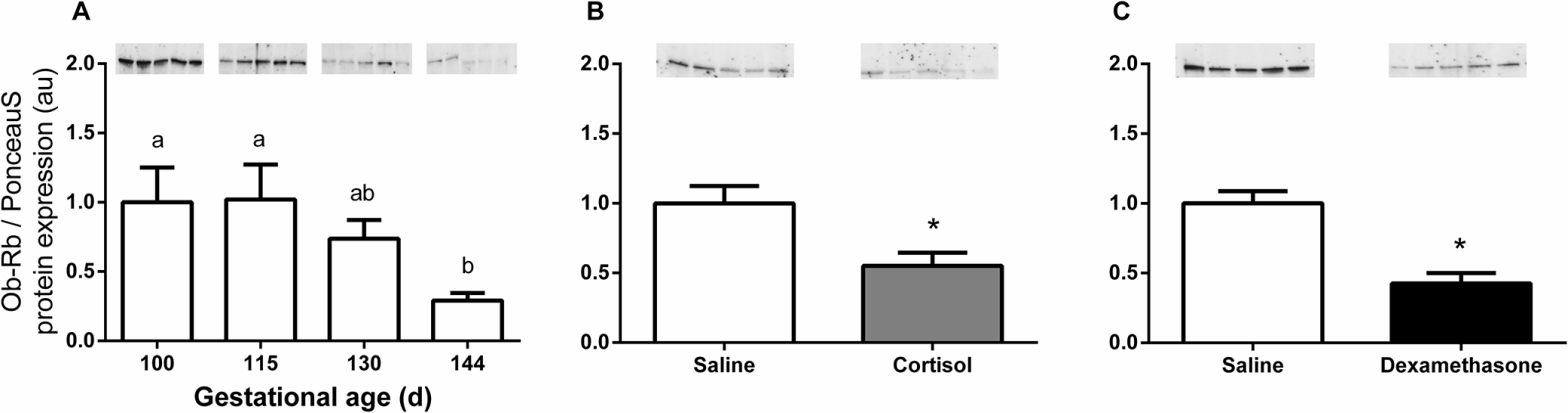
Mean (± SEM) relative mRNA abundance, normalised to respective control saline group, of leptin receptors (Ob-Rb and Ob-R) in the lungs of sheep fetuses exposed to glucocorticoids by fetal cortisol infusion (A, B) and maternal dexamethasone treatment (C, D; n = 5–6 for each group). * significantly different from respective control saline group, P<0.05, unpaired t-test.

Pulmonary Ob-Rb mRNA levels in the cortisol-infused fetuses were significantly greater than those in the saline-infused fetuses (2.3 fold, P<0.05, Fig 4A), but mRNA abundance of Ob-R (all forms) remained unchanged (Fig 4B). Maternal dexamethasone treatment caused significant increases in both pulmonary Ob-Rb (4.3 fold, P<0.005, Fig 4C) and Ob-R transcripts (all forms; 2.3 fold, P<0.005, Fig 4D). Lung Ob-Rb protein content was significantly reduced by both fetal cortisol infusion and maternal dexamethasone administration (P<0.05, Figs 3B and 4C).

No significant changes in pulmonary protein levels of pSTAT3, STAT3 or pSTAT3:STAT3 ratio were observed with increasing gestational age or glucocorticoid treatment (Table 3). Leptin mRNA was not detected in the fetal ovine lungs at any of the gestational ages studied or after glucocorticoid treatment.

**Table 3:** Mean (± SEM) relative protein levels of pulmonary pSTAT3, STAT3 and pSTAT3:STAT3 ratio in untreated ovine fetuses between 100 and 144d of gestation, and in glucocorticoid-treated ovine fetuses at 127–130d of gestation (n = 5–6 in each group). Data are normalised to 100d gestational age group or respective saline control group, as appropriate. Au, arbitrary units.

	pSTAT3 (au)	STAT3 (au)	pSTAT3:STAT3 (au)
**Developmental study**			
100d (n = 5)	1.00 ± 0.12	1.00 ± 0.10	1.00 ± 0.04
115d (n = 5)	0.91 ± 0.12	0.81 ± 0.15	1.21 ± 0.13
130d (n = 5)	1.01 ± 0.12	0.94 ± 0.09	1.09 ± 0.10
144d (n = 5)	0.67 ± 0.10	0.73 ± 0.12	0.94 ± 0.09
**Fetal cortisol study**			
Saline 127–130d (n = 6)	1.00 ± 0.17	1.00 ± 0.11	1.00 ± 0.09
Cortisol 127–130d (n = 5)	0.86 ± 0.18	0.92 ± 0.11	0.94 ± 0.14
**Maternal dexamethasone study**			
Saline 127–130d (n = 5)	1.00 ± 0.10	1.00 ± 0.10	1.00 ± 0.07
Dexamethasone 127–130d (n = 5)	1.09 ± 0.28	1.01 ± 0.15	0.99 ± 0.16

### Relationships between pulmonary Ob-Rb mRNA and protein levels, and plasma hormone concentrations

In the untreated fetuses studied from 100 to 144d of gestation, significant positive correlations were observed between plasma leptin and plasma concentrations of cortisol (r = 0.54, n = 20, P<0.005) and T_3_ (r = 0.61, n = 20, P<0.005), but not T_4_. Significant positive relationships were identified between pulmonary Ob-Rb mRNA abundance and plasma concentrations of cortisol (r = 0.78, n = 19, P<0.05) and T_3_ (r = 0.63, n = 19, P<0.05), but not plasma leptin or T_4_. Pulmonary leptin Ob-R mRNA (all forms) was significantly related to plasma concentrations of cortisol (r = 0.65, n = 20, P<0.05) and T_3_ (r = 0.55, n = 20, P<0.05), but not T_4_. Significant negative correlations were observed between mRNA abundance and protein levels of Ob-Rb in the fetal lungs (r = 0.60, n = 19, P<0.05). Pulmonary Ob-Rb protein levels were inversely related to plasma concentrations of cortisol (r = -0.65, n = 20, P<0.05) and T_3_ (r = -0.46, n = 20, P<0.05), but not plasma leptin or T_4_ concentrations.

## Discussion

This study is the first to report changes in the leptin receptor and its transcript, specifically the fully active long-form receptor Ob-Rb, in the fetal ovine lung during late gestation and in response to increased glucocorticoid exposure *in utero*. With advancing gestational age and treatment with endogenous and synthetic glucocorticoids, pulmonary Ob-Rb mRNA levels increased, and yet Ob-Rb protein content decreased, in the sheep fetus. These findings support, in part, those observed in the baboon fetus where pulmonary Ob-Rb mRNA abundance increased from 0.34 to 0.89 fraction of gestational age (equivalent to 50d to 130d of gestation in the sheep fetus) and Ob-R protein was detected only at the late stage of gestation [15]. In addition, a rise in Ob-Rb mRNA levels has been shown to occur between embryonic days 17 (0.79) and 20 (0.93) in the lungs of fetal rats [27]. The differential effects of glucocorticoids *in utero* on the Ob-Rb transcript and protein in the fetal ovine lungs suggest regulation of transcript level, translation, and/or post-translational processing. Indeed, a lack of correlation between mRNA and protein levels for Ob-Rb has been reported previously in the uterus of pigs during the oestrous cycle and early pregnancy [28]. Furthermore, an increase in Ob-Rb mRNA abundance is observed without any change in Ob-Rb protein content in cultured adrenocortical cells taken from adult sheep exposed to the synthetic glucocorticoid betamethasone *in utero* [29].

The mechanisms responsible for the developmental and glucocorticoid-control of pulmonary Ob-Rb content may be direct and/or indirectly involve other endocrine systems. In the present study, the changes in Ob-Rb and its transcript seen in the fetal lungs were associated with increased glucocorticoid activity and elevated plasma concentrations of T_3_ and leptin [8, 14, 30]. Maternal dexamethasone treatment has also been shown previously to increase plasma insulin concentration in these sheep fetuses [31]. Putative glucocorticoid and thyroid hormone response elements have been identified in 2,000-bp upstream of the 5’-region of exon 1 in the chicken leptin receptor gene [32], and glucocorticoids, T_3_ and insulin upregulate Ob-Rb mRNA levels independently in a variety of cell types *in vitro* [33, 34, 35, 36]. Furthermore, in rats, elevating fetal corticosterone concentration by maternal nicotine or caffeine treatment is associated with dose-dependent increments in Ob-Rb mRNA in the fetal liver [37, 38]. Leptin itself is also able to modulate Ob-Rb expression in a negative feedback mechanism. In common with hormones and other ligands that bind to cell surface receptors, leptin binding to the leptin receptor causes internalisation and lysosomal degradation of the hormone, and down-regulation of the receptor [39]. Therefore, high circulating leptin concentration may contribute to the lower pulmonary Ob-Rb protein levels, and, in turn, maintenance of the pSTAT3:STAT3 ratio and JAK/STAT signalling, seen in the sheep fetuses exposed to glucocorticoids. Ontogenic and glucocorticoid-induced changes in the internalisation and trafficking of the leptin receptor in the developing lungs or other tissues, however, are unknown.

In addition to the Ob-Rb, leptin can bind to at least five other leptin receptor isoforms [5]. These receptors, including the soluble leptin receptor Ob-Re, buffer free leptin concentrations and facilitate leptin transport in the circulation and within tissues [5]. In the present study, pulmonary Ob-R mRNA levels, detected with the probe that recognises all forms of the leptin receptor, increase towards term in fetal sheep and in response to maternal dexamethasone treatment, but not fetal cortisol infusion. The developmental and glucocorticoid-induced changes in pulmonary Ob-Rb mRNA abundance contribute, in part, to these findings, but it is possible that there may be coincident effects on the other isoforms of leptin receptor, with important consequences for the bioavailability of leptin *in utero*. For example, there may be a reduction in the gene expression of other Ob-R isoforms in the sheep fetuses infused with cortisol. Furthermore, the functionality of the Ob-Rb depends upon the components of the intracellular signalling pathways, which may show developmental and glucocorticoid-sensitive changes in the lungs of the sheep fetus. Indeed, in the present study, the phosphorylation status of STAT3 in the ovine fetal lungs appeared to be unaffected by gestational age and glucocorticoid treatment, despite reductions in pulmonary Ob-Rb protein content. The JAK/STAT pathway is known to be influenced by growth factors and cytokines other than leptin, and in a human lung adenocarcinoma cell line, dexamethasone has been shown to enhance interleukin-6 activation of JAK/STAT signalling without any direct effect on pSTAT3 (Ser727 or Tyr705) or STAT3 protein concentration [40].

The cellular localisation of Ob-Rb protein in the fetal ovine lungs is similar to that previously reported in other species both before and after birth [15, 41]. The widespread presence of Ob-Rb protein in the pulmonary epithelium of the sheep fetus from at least 100d of gestation suggests that leptin may have a role in promoting lung growth *in utero*. Previous studies have demonstrated that treatment of pregnant mice with leptin causes an increase in the lung weight of the fetus [27, 42]. In addition, leptin stimulates cell proliferation in a human lung squamous cell line, and in a primary cell culture of tracheal epithelial cells from wild-type mice but not *db/db* mice with mutated leptin receptors [43]. The ontogenic decrease in Ob-Rb protein levels in the fetal ovine lungs coincides with progression from the canalicular (80–120d) to saccular (120d to term) and alveolar (120d to postnatal life) stages of lung development [44]. During these stages, the pulmonary epithelium becomes thinner and differentiates into distinct populations of type I and II pneumocytes [44]. Near term, maturational changes in the structure and function of the fetal pulmonary epithelium are stimulated by the prepartum cortisol surge [13]. It is possible, therefore, that leptin acts as a stimulus for general lung growth before birth and that the developmental and glucocorticoid-induced suppression of pulmonary Ob-Rb contributes to the maturational shift towards alveolar thinning and pneumocyte differentiation. Alternatively, localisation of Ob-Rb protein to type II pneumocytes in the pulmonary epithelium and an overall reduction in lung Ob-Rb protein content seen in the fetuses exposed to endogenous and exogenous glucocorticoids may be a consequence, rather than cause, of pneumocyte differentiation. Furthermore, maintenance of pulmonary pSTAT3:STAT ratio in fetuses with lower Ob-Rb protein may indicate an increase in cell-specific leptin signalling during lung maturation.

Localisation of Ob-Rb protein to type II pneumocytes in fetal ovine lungs, especially in the fetuses at 144d of gestation, supports a role for leptin in the activation of surfactant production near term. In fetal rat lung explants, physiological concentrations of leptin increase mRNA abundance of surfactant proteins A, B and C and protein levels of surfactant proteins A and B [18, 42]. Leptin also increases the synthesis of surfactant phospholipids in fetal rat lung explants and in isolated pneumocytes from fetal rabbits [18, 41]. In addition, antenatal treatment of pregnant rats with leptin increases type II pneumocyte number and surfactant protein expression in the fetal lungs [27, 42]. In contrast, leptin had no effect on lung compliance, phospholipid content or surfactant protein expression in a study using fetal sheep *in vivo* [45]. However, this study used ultrasound-guided intramuscular leptin injection and circulating concentrations of leptin *in utero* were not determined. Intravenous leptin infusion for five days in fetal sheep promotes aspects of lung maturation, including increased pulmonary surfactant protein B mRNA abundance [46]. Leptin receptor protein was also evident in alveolar macrophages in the fetal ovine lungs near term and leptin may play a role in the development of pulmonary immune defence mechanisms. Alveolar macrophages taken from leptin-deficient adult mice show impaired phagocytosis and leukotriene synthesis *in vitro* which can be restored to normal by exogenous leptin treatment [47]. Physiological doses of leptin can also stimulate leukotriene synthesis in alveolar macrophages obtained from normal adult rat lungs [48].

Leptin receptor protein was also localised to the chondrocytes of bronchial cartilage in the fetal ovine lungs. Previous studies have demonstrated the presence of leptin receptor mRNA and protein in developing cartilage-bone in the mouse fetus [16]. In cell culture of human articular chondrocytes, leptin has been shown to stimulate chondrocyte proliferation and differentiation, and synthesis of collagen and proteoglycans in the extracellular matrix [49]. Therefore, leptin may be involved in the growth and development of pulmonary epithelium, type II pneumocytes, macrophages and bronchial cartilage *in utero*. The absence of leptin mRNA in the fetal ovine lungs at the gestational ages studied, however, suggests that the developmental and glucocorticoid-induced actions of leptin via Ob-Rb are likely to be endocrine rather than by local synthesis and paracrine actions of leptin. There appear to be species differences in pulmonary leptin synthesis before birth as leptin mRNA abundance has been detected in the lungs of fetal rats which increases with gestational age, and other studies have proposed paracrine interactions between leptin and parathyroid-hormone related protein in the control of surfactant production in the rat fetus [18, 27]. The presence of leptin receptors in the fetal lungs and their potential roles in pulmonary growth and maturation may also have consequences for respiratory function that extend into neonatal and adult life. Alveolar development is impaired in leptin-deficient *ob/ob* mice from the earliest time point studied at two weeks of postnatal life [50]. The contribution that leptin makes to glucocorticoid-induced lung maturation and the extent to which respiratory disorders in neonatal and adult life have their origins in leptin dysfunction before birth remains to be established.

## Supporting Information

S1 File Primary Data. (XLSX)
